# Supplementary material for: Combination of Muscle Quantity and Quality Is Useful to Assess the Necessity of Surveillance after a 5-Year Cancer-Free Period in Patients Who Undergo Radical Cystectomy: A Multi-Institutional Retrospective Study
Source: Cancers (Basel). 2023 Feb 27;15(5):1489. doi: 10.3390/cancers15051489 (PMC10000682; doi:10.3390/cancers15051489)
Supplement: Supplementary file 1 [file cancers-15-01489-s001.zip › cancers-2129246-supplementary.pdf]

Supplementary Table S1. Univariable analyses for recurrence-free survival.

	Factor	*P* value	Hazard ratio	95% CI
Age	Continuous	0.405	1.033	0.957–1.114
Sex	Male	0.369	2.600	0.324–20.89
Neoadjuvant chemotherapy	Received	0.688	1.314	0.347–4.972
Urinary diversion	Ileal neobladder	0.956	0.962	0.240–3.854
Histology	Pure UC	0.569	1.830	0.229–14.64
Pathological T stage	pT0	0.207	2.483	0.604–10.20
Pathological T stage	pT3 or pT4	0.348	0.035	0.000–38.69
Pathological N stage	pN1–3	0.578	1.814	0.223–14.76
Tumor grade	Grade 3	0.838	1.178	0.245–5.674
Lymphovascular invasion	Positive	0.502	0.583	0.121–2.816
Adjuvant chemotherapy	Received	0.443	0.039	0.000–157.7

CI, confidence interval; UC, urothelial carcinoma.
